# Supplementary material for: A Critical View of the Application of the APEX Software (Aqueous Photochemistry of Environmentally-Occurring Xenobiotics) to Predict Photoreaction Kinetics in Surface Freshwaters
Source: Molecules. 2019 Dec 18;25(1):9. doi: 10.3390/molecules25010009 (PMC7017383; doi:10.3390/molecules25010009)
Supplement: Supplementary file 1 [file molecules-25-00009-s001.zip › 3.2.4_gcc-4.4.0.zip!/3.2.4_gcc-4.4.0/3.2.4_gcc-4.4.0/doc/octave/html/interpreter/Special-Utility-Matrices.html]

Special Utility Matrices - Untitled


Next: Famous Matrices,
Previous: Applying a Function to an Array,
Up: Matrix Manipulation


---

### 16.4 Special Utility Matrices

— Built-in Function: **eye** (x)  
— Built-in Function: **eye** (n, m)  
— Built-in Function: **eye** (..., class)  
> Return an identity matrix. If invoked with a single scalar argument,
> `eye` returns a square matrix with the dimension specified. If you
> supply two scalar arguments, `eye` takes them to be the number of
> rows and columns. If given a vector with two elements, `eye` uses
> the values of the elements as the number of rows and columns,
> respectively. For example,
>
> ```
>           eye (3)
>                =>  1  0  0
>                    0  1  0
>                    0  0  1
> ```
>
> The following expressions all produce the same result:
>
> ```
>           eye (2)
>           ==
>           eye (2, 2)
>           ==
>           eye (size ([1, 2; 3, 4])
> ```
>
> The optional argument class, allows `eye` to return an array of
> the specified type, like
>
> ```
>           val = zeros (n,m, "uint8")
> ```
>
> Calling `eye` with no arguments is equivalent to calling it
> with an argument of 1. This odd definition is for compatibility
> with matlab.

— Built-in Function: **ones** (x)  
— Built-in Function: **ones** (n, m)  
— Built-in Function: **ones** (n, m, k, ...)  
— Built-in Function: **ones** (..., class)  
> Return a matrix or N-dimensional array whose elements are all 1.
> The arguments are handled the same as the arguments for `eye`.
>
> If you need to create a matrix whose values are all the same, you should
> use an expression like
>
> ```
>           val_matrix = val * ones (n, m)
> ```
>
> The optional argument class, allows `ones` to return an array of
> the specified type, for example
>
> ```
>           val = ones (n,m, "uint8")
> ```

— Built-in Function: **zeros** (x)  
— Built-in Function: **zeros** (n, m)  
— Built-in Function: **zeros** (n, m, k, ...)  
— Built-in Function: **zeros** (..., class)  
> Return a matrix or N-dimensional array whose elements are all 0.
> The arguments are handled the same as the arguments for `eye`.
>
> The optional argument class, allows `zeros` to return an array of
> the specified type, for example
>
> ```
>           val = zeros (n,m, "uint8")
> ```

— Function File: **repmat** (A, m, n)  
— Function File: **repmat** (A, [m n])  
— Function File: **repmat** (A, [m n p ...])  
> Form a block matrix of size m by n, with a copy of matrix
> A as each element. If n is not specified, form an
> m by m block matrix.

— Loadable Function: **rand** (x)  
— Loadable Function: **rand** (n, m)  
— Loadable Function: **rand** ("state", x)  
— Loadable Function: **rand** ("seed", x)  
> Return a matrix with random elements uniformly distributed on the
> interval (0, 1). The arguments are handled the same as the arguments
> for `eye`.
>
> You can query the state of the random number generator using the
> form
>
> ```
>           v = rand ("state")
> ```
>
> This returns a column vector v of length 625. Later, you can
> restore the random number generator to the state v
> using the form
>
> ```
>           rand ("state", v)
> ```
>
> You may also initialize the state vector from an arbitrary vector of
> length <= 625 for v. This new state will be a hash based on the
> value of v, not v itself.
>
> By default, the generator is initialized from `/dev/urandom` if it is
> available, otherwise from cpu time, wall clock time and the current
> fraction of a second.
>
> To compute the pseudo-random sequence, `rand` uses the Mersenne
> Twister with a period of 2^19937-1 (See M. Matsumoto and T. Nishimura,
> Mersenne Twister: A 623-dimensionally equidistributed uniform pseudorandom number generator, ACM Trans. on
> Modeling and Computer Simulation Vol. 8, No. 1, January pp.3-30 1998,
> http://www.math.sci.hiroshima-u.ac.jp/~m-mat/MT/emt.html).
> Do **not** use for cryptography without securely hashing
> several returned values together, otherwise the generator state
> can be learned after reading 624 consecutive values.
>
> Older versions of Octave used a different random number generator.
> The new generator is used by default
> as it is significantly faster than the old generator, and produces
> random numbers with a significantly longer cycle time. However, in
> some circumstances it might be desirable to obtain the same random
> sequences as used by the old generators. To do this the keyword
> "seed" is used to specify that the old generators should be use,
> as in
>
> ```
>           rand ("seed", val)
> ```
>
> which sets the seed of the generator to val. The seed of the
> generator can be queried with
>
> ```
>           s = rand ("seed")
> ```
>
> However, it should be noted that querying the seed will not cause
> `rand` to use the old generators, only setting the seed will.
> To cause `rand` to once again use the new generators, the
> keyword "state" should be used to reset the state of the `rand`.
>
>
>
> **See also:** randn, rande, randg, randp.

— Loadable Function: **randn** (x)  
— Loadable Function: **randn** (n, m)  
— Loadable Function: **randn** ("state", x)  
— Loadable Function: **randn** ("seed", x)  
> Return a matrix with normally distributed pseudo-random
> elements having zero mean and variance one. The arguments are
> handled the same as the arguments for `rand`.
>
> By default, `randn` uses the Marsaglia and Tsang “Ziggurat technique” to
> transform from a uniform to a normal distribution. (G. Marsaglia and
> W.W. Tsang, Ziggurat method for generating random variables,
> J. Statistical Software, vol 5, 2000,
> http://www.jstatsoft.org/v05/i08/)
>
>
>
> **See also:** rand, rande, randg, randp.

— Loadable Function: **rande** (x)  
— Loadable Function: **rande** (n, m)  
— Loadable Function: **rande** ("state", x)  
— Loadable Function: **rande** ("seed", x)  
> Return a matrix with exponentially distributed random elements. The
> arguments are handled the same as the arguments for `rand`.
>
> By default, `randn` uses the Marsaglia and Tsang “Ziggurat technique” to
> transform from a uniform to a exponential distribution. (G. Marsaglia and
> W.W. Tsang, Ziggurat method for generating random variables,
> J. Statistical Software, vol 5, 2000,
> http://www.jstatsoft.org/v05/i08/)
>
>
>
> **See also:** rand, randn, randg, randp.

— Loadable Function: **randp** (l, x)  
— Loadable Function: **randp** (l, n, m)  
— Loadable Function: **randp** ("state", x)  
— Loadable Function: **randp** ("seed", x)  
> Return a matrix with Poisson distributed random elements with mean value parameter given by the first argument, l. The arguments
> are handled the same as the arguments for `rand`, except for the
> argument l.
>
> Five different algorithms are used depending on the range of l
> and whether or not l is a scalar or a matrix.
>
> For scalar l <= 12, use direct method.: Press, et al., 'Numerical Recipes in C', Cambridge University Press, 1992. For scalar l > 12, use rejection method.[1]: Press, et al., 'Numerical Recipes in C', Cambridge University Press, 1992. For matrix l <= 10, use inversion method.[2]: Stadlober E., et al., WinRand source code, available via FTP. For matrix l > 10, use patchwork rejection method.: Stadlober E., et al., WinRand source code, available via FTP, or H. Zechner, 'Efficient sampling from continuous and discrete unimodal distributions', Doctoral Dissertation, 156pp., Technical University Graz, Austria, 1994. For l > 1e8, use normal approximation.: L. Montanet, et al., 'Review of Particle Properties', Physical Review D 50 p1284, 1994
>
>
>
>
> **See also:** rand, randn, rande, randg.

— Loadable Function: **randg** (a, x)  
— Loadable Function: **randg** (a, n, m)  
— Loadable Function: **randg** ("state", x)  
— Loadable Function: **randg** ("seed", x)  
> Return a matrix with `gamma(`a`,1)` distributed random elements.
> The arguments are handled the same as the arguments for `rand`,
> except for the argument a.
>
> This can be used to generate many distributions:
>
> `gamma (a, b)` for `a > -1`, `b > 0`: ``` r = b * randg (a) ``` `beta (a, b)` for `a > -1`, `b > -1`: ``` r1 = randg (a, 1) r = r1 / (r1 + randg (b, 1)) ``` `Erlang (a, n)`: ``` r = a * randg (n) ``` `chisq (df)` for `df > 0`: ``` r = 2 * randg (df / 2) ``` `t(df)` for `0 < df < inf` (use randn if df is infinite): ``` r = randn () / sqrt (2 * randg (df / 2) / df) ``` `F (n1, n2)` for `0 < n1`, `0 < n2`: ``` ## r1 equals 1 if n1 is infinite r1 = 2 * randg (n1 / 2) / n1 ## r2 equals 1 if n2 is infinite r2 = 2 * randg (n2 / 2) / n2 r = r1 / r2 ``` negative `binomial (n, p)` for `n > 0`, `0 < p <= 1`: ``` r = randp ((1 - p) / p * randg (n)) ``` non-central `chisq (df, L)`, for `df >= 0` and `L > 0`: (use chisq if `L = 0`) ``` r = randp (L / 2) r(r > 0) = 2 * randg (r(r > 0)) r(df > 0) += 2 * randg (df(df > 0)/2) ``` `Dirichlet (a1, ... ak)`: ``` r = (randg (a1), ..., randg (ak)) r = r / sum (r) ```
>
>
>
>
> **See also:** rand, randn, rande, randp.

The generators operate in the new or old style together, it is not
possible to mix the two. Initializing any generator with
`"state"` or `"seed"` causes the others to switch to the
same style for future calls.

The state of each generator is independent and calls to different
generators can be interleaved without affecting the final result. For
example,

```
     rand ("state", [11, 22, 33]);
     randn ("state", [44, 55, 66]);
     u = rand (100, 1);
     n = randn (100, 1);
```

and

```
     rand ("state", [11, 22, 33]);
     randn ("state", [44, 55, 66]);
     u = zeros (100, 1);
     n = zeros (100, 1);
     for i = 1:100
       u(i) = rand ();
       n(i) = randn ();
     end
```

produce equivalent results. When the generators are initialized in
the old style with `"seed"` only `rand` and `randn` are
independent, because the old `rande`, `randg` and
`randp` generators make calls to `rand` and `randn`.

The generators are initialized with random states at start-up, so
that the sequences of random numbers are not the same each time you run
Octave.^1^ If you really do
need to reproduce a sequence of numbers exactly, you can set the state
or seed to a specific value.

If invoked without arguments, `rand` and `randn` return a
single element of a random sequence.

The original `rand` and `randn` functions use Fortran code from
Ranlib, a library of fortran routines for random number generation,
compiled by Barry W. Brown and James Lovato of the Department of
Biomathematics at The University of Texas, M.D. Anderson Cancer Center,
Houston, TX 77030.

— Function File: **randperm** (n)  
> Return a row vector containing a random permutation of the
> integers from 1 to n.

The functions `linspace` and `logspace` make it very easy to
create vectors with evenly or logarithmically spaced elements.
See Ranges.

— Built-in Function: **linspace** (base, limit, n)  
> Return a row vector with n linearly spaced elements between
> base and limit. If the number of elements is greater than one,
> then the base and limit are always included in
> the range. If base is greater than limit, the elements are
> stored in decreasing order. If the number of points is not specified, a
> value of 100 is used.
>
> The `linspace` function always returns a row vector.
>
> For compatibility with matlab, return the second argument if
> fewer than two values are requested.

— Function File: **logspace** (base, limit, n)  
> Similar to `linspace` except that the values are logarithmically
> spaced from
> 10^base to 10^limit.
>
> If limit is equal to
> pi,
> the points are between
> 10^base and pi,
> *not*
> 10^base and 10^pi,
> in order to be compatible with the corresponding matlab
> function.
>
> Also for compatibility, return the second argument if fewer than two
> values are requested.
>
>
>
> **See also:** linspace.

---

#### Footnotes

[1] The old versions of `rand` and `randn`
obtain their initial seeds from the system clock.

---
